# Outsourcing Elderly Care to Migrant Workers: The Impact of Gender and Class on the Experience of Male Employers

**DOI:** 10.1177/0038038515573688

**Published:** 2015-04-28

**Authors:** Ester Gallo, Francesca Scrinzi

**Affiliations:** Gediz University, Turkey; University of Glasgow, UK

**Keywords:** class, employers, family, gender, masculinity, men, migrant care workers

## Abstract

This article, based on semi-structured interviews, addresses masculinity in the international division of reproductive labour through an analysis of the impact of gender and class on the outsourcing of elderly care services to migrant care workers. In the Italian context, characterised by a limited provision of long-term care services and by cash-for-care benefits, the strategies of men as employers of migrant care workers are shaped by class and gender. The outsourcing of care to migrant workers reproduces hegemonic masculinity in so far as male employers are able to withdraw from the ‘dirty work’. At the same time, men engage with tasks which are, in principle, kept at a distance. The employers’ family status, combined with their class background, are crucial factors in shaping the heterogeneity of men’s experiences as employers and managers of care labour, and the ways in which they make sense of their masculinity.

This article aims to broaden our understanding of the international division of reproductive labour^1^ (henceforth IDRL) by analysing the experiences of male employers with different class backgrounds. By ‘employers’ we mean those individuals who are responsible for recruiting, managing and supervising a worker who provides care for an elderly relative (usually a parent, spouse or sibling). We consider employers as ‘informal care providers’ (Kramer, 2002: 6–7): those subjects who, being required to engage with the physical or psychological needs of others, experience changes in their accustomed roles, social relations and self-perceptions.

We consider class, gender and ethnicity as primary social divisions in contemporary societies, based on and reproducing both symbolic hierarchies and material inequalities in resource allocation and consumption (Anthias, 2001). Holding that ‘class is not an economic relation per se’, we investigate how both ‘class effects’ and ‘economic effects’ (Anthias, 2001: 846) shape our informants’ lives. Income – combined with access to cash benefits – conditions families’ access to full/part-time services and the management of migrant care labour. Class is also differentiated because of its interplay with gender and ethnicity and in relation to occupational cultures: the outsourcing of care to migrant workers reflects and moulds class differences, contributing to the construction of a variety of gendered and racialised models of filial duty and conjugality. While ethnicity is highly significant in shaping the employers’ practices in the IDRL, we consider here how gender and class are shaped through the outsourcing of care to migrant workers without focusing on processes of racialisation, choosing to develop this area in other publications. Our analysis draws from semi-structured interviews conducted in urban Italy among male employers over six years. We explore how masculinities are constructed through the consumption of home-based care services for elderly people, and how notions of family relations are reworked in the process. We suggest that men’s engagement with care labour as employers plays an important role in the construction of models of masculinity which are differentiated across class. We argue that family status and kinship relations with the care beneficiary are key in appreciating the shifting gendered division of work in the family and how male employers engage with migrant care labour.

The article makes two original contributions. First, it enhances our understanding of the IDRL by investigating the employers’ role in shaping the demand for flexible migrant labour. While the employers’ role in driving the demand for foreign-born workers is considered a key issue in the social sciences, it remains largely under-researched and under-theorised (Cangiano and Walsh, 2014; Hondagneu-Sotelo, 2001; McGovern, 2007; Mahler and Pessar, 2006; Näre, 2013b; Rodriguez, 2004; Waldinger and Lichter, 2003). Second, the article contributes to a more relational understanding of gender and globalisation by considering men’s practices and the social construction of masculinities in relation to the management of household-based care services. Focusing on male employers of different class backgrounds enables us to de-centre the attention predominantly paid to how hegemonic masculinity is constructed in relation to prestigious careers (Connell, 1998) and goes beyond the essentialist conceptualisation of paid domestic/care work relationships as ‘women’s business’.

We begin by discussing the need to adopt a more relational approach to the gendered employer–employee relationship within the IDRL and link this to emerging work on masculinity and care. After discussing the Italian context and our methodological strategy, we explore the role of family status and kinship relations in moulding men’s experiences as employers between withdrawal and progressive involvement in the ‘dirty work’ of care management.

## Employing Care Workers: Gender, Ethnicity and Class Dynamics

Recent scholarship has addressed the care/domestic work sector as a crucial context in which to analyse the emergence of transnational gendered and ethnic hierarchies against the backdrop of neoliberal economies and welfare states (Andall, 2000; Anderson, 2000; Hochschild, 2000; Hondagneu-Sotelo, 2001; Parreñas, 2001). This sector – which in 2010 accounted for 3.2 per cent of worldwide employment and involves today nearly 52.6 million women and men (ILO, 2011) – has witnessed an increasing ‘migrantisation’ (Kilkey et al., 2010: 380): migrant labour now functions as an alternative to direct state care service provision (Bettio et al., 2006; Huang et al., 2012; Kilkey et al., 2010; Lutz, 2008; Sciortino, 2004). A ‘three tier transfer’ (Parreñas, 2001: 561) within the IDRL materialises through privileged women purchasing low-wage domestic services from migrant women who, in turn, employ lower-wage services in their own home countries to look after their families left behind.

The ‘employer demand for labour is a powerful tool for understanding gendered employment patterns’ and social mobility of migrants (Mahler and Pessar, 2006: 46). Yet the ‘globalisation of care’ has been mainly conceptualised in terms of a ‘female employer–female employee’ relationship, privileging a focus on the experiences of migrant employees (Andall, 2000; Ehrenreich and Hochschild, 2002; Lutz, 2002) as opposed to those of their employers. Limited studies show how employer/employee relations are structured around class and racism (Glenn, 1992; Hondagneu-Sotelo, 2001; Moras, 2008; Palmer, 1990) and how they reproduce dominant and racialised femininities (Anderson, 2007; Rollins, 1985; Scrinzi, 2013; Uttal and Tuominen, 1999) without effectively challenging the gendered division of work in the employers’ household (Anderson, 2000).

By focusing on upper-middle-class households, this literature unravels how the construction of gender in privileged households is based on the outsourcing of care/domestic work to migrants (Gregson and Lowe, 1994; Hondagneu-Sotelo, 2001; Kilkey et al., 2013; Lan, 2006). But not all employers belong to a ‘high achieving and time-pressed’ upper middle class (Lundstrom, 2012: 153). Class and ethnicity intersect in driving care service consumption, as employers have different access to migrant labour according to their economic means (Näre, 2013b; Triandafyllidou and Marchetti, 2014). Indeed, in countries like Italy or the USA, employers also increasingly come from the working and lower middle classes (Sarti, 2008; Williams, 2010). Scholars have largely interpreted this shift in terms of decreasing importance of class status in structuring the demand for (migrant) care work, noting how, while the demand for paid domestic work is closely connected to an upper-middle-class lifestyle, class status issues are less important in moulding the consumption of elderly care labour provided by migrants (Da Roit, 2007; Näre, 2013a). On the basis of our data, however, we claim that both income and status are central in shaping families’ access to migrant care labour. Further, we argue that the call for a more relational understanding of gender within the IDRL (Kofman and Raghuram, 2007; Yeates, 2009) requires scholars to engage with the diversification of the demand-side across class and gender and with men’s *active* role in care labour consumption.

Limited work on masculinity within the IDRL notes how migrant men’s employment as care/domestic workers challenges the conventional association of these jobs with female labour, identifying them as a site where hegemonic and subaltern masculinities are produced (Chopra, 2006; Gallo, 2006; Kilkey, 2010; Sarti, 2006; Scrinzi, 2010). More broadly, this work speaks to the recent interest in masculinities within ‘feminine occupational cultures’. It unravels how men appropriate occupational resources once seen as particular to women to resist gendered stereotypes and enhance their careers (Hall et al., 2007; Williams, 1995).

Pioneering work on male employers within the IDRL has also noted how upper-middle-class men often come to play an agentive role in driving demand for commoditised domestic services. Kilkey et al.’s (2013) work on young professional couples employing migrant handymen in the UK and Europe highlights how this labour is functional to the attainment of new models of upper-middle-class ‘nurturing fathers’ involved in caring and leisure activities with their children. Cox (2006) indicates that single male professionals living alone or with male friends are more likely to hire cleaners than single women.

From a different perspective, scholarship on masculinity has shown how care is crucial in nuancing men’s experiences in contemporary societies (Calasanti and King, 2007; Russell, 2007; Thompson, 2002). Although women in the family still perform a significantly higher share of care work (Saraceno, 2010), men are increasingly more involved in unpaid family care, according to class differences. While upper- and middle-class families are characterised by more liberal gender ideologies than working-class families, the gendered division of work tends to be more egalitarian in the latter: here it is associated with lower education levels, economic precariousness and the lack of outsourcing strategies which are available to better-off families (Shows and Gerstel, 2009). In Europe, working-class individuals are more likely to be personally involved in providing care to their elderly relatives than those with higher education levels (Saraceno, 2010). As Hanlon (2012: 6) notes, care relations are a ‘source of tensions and contradictions in men’s lives’ as they require men to negotiate between ‘hegemonic dictates of masculinity’ and the practices, knowledge and emotions involved in the necessity to engage with care.

Both the scholarship on men in the IDRL and on men as carers offer insight into how masculinity is constructed around the provision or consumption of household-based services, and counterbalance the long-standing focus on masculinity within managerial careers (Acker, 2004; Hacker, 1989; Jackall, 1988). They invite scholars to go beyond a fixed and trans-historical model of hegemonic masculinity and to challenge existing dichotomies between women’s and men’s experiences of globalisation (Connell and Messerschmidt, 2005; Poster, 2002).

Drawing from this scholarship, we argue that the household should be analysed as an important site where global – not just hegemonic and racialised but also subaltern (including working-class) – masculinities (Connell, 1998) are forged through the enactment of family and work relations. Although within the IDRL women are assigned a major responsibility in managing domestic/care workers, men too can be involved in these interactions. Men are not simply the (material and symbolic) beneficiaries of paid care/domestic work but also gendered social actors who develop strategies to maintain their material and symbolic privileges in order to accommodate changing gender relations. In doing so, they actively contribute not only to shape the domestic/care service relationship, but also to transform masculinity (and femininity). We argue that the outsourcing of care is crucial in understanding how gendered family relations are reworked in neoliberal welfare regimes and in capturing men’s experiences in relation to their roles as sons, husbands or brothers.

Our work departs from existing research in two respects. First, we move beyond an exclusive focus on upper-middle-class employers and explore how white-collar and working-class masculinities are forged in the household as this becomes a workplace for migrant care-givers. Second, we note how current studies often implicitly assume that men consume home-based paid care labour mainly as married subjects or in relation to female partners, and downplay those situations in which single, divorced or widowed men cope with the care of their elderly parents without the support of a female relative. Instead, as our data show, the employment of a care-giver can be a crucial process in the construction and negotiation of men’s identities beyond normative models of conjugality.

## Migration, the Welfare State and the Demand for Care Labour in Italy

Italy well exemplifies trends affecting European countries regarding the interconnections between welfare systems, gender regimes, care models and international migration. Italy has one of the highest rates of elderly inhabitants in Europe: 20.8 per cent of the national population are aged over 65. Available statistics indicate a growing structural dependency of over-65s on the active population (to 32 per cent in 2012) and that nearly 40 per cent of the elderly population require assistance (INPS, 2012; ISTAT, 2012). Italy is seen as epitomising a ‘Mediterranean pattern’ where a familialistic welfare state system delegates to families (particularly to women) the burden of elderly care (Lyon and Glucksmann, 2008; Näre, 2013b). Yet, as in Northern European countries, the Italian system increasingly operates through cash transfers to families, mainly in the form of pensions but also through the payment of attendance allowances for dependent/disabled persons (Anderson, 2007; Bettio and Plantenga, 2004; Williams, 2010). Cash benefits (around 500 Euros monthly) have grown throughout the 2000s; in 2012 in some regions the share of the entitled elderly population was 12.5 per cent, reaching 19 per cent (INPS, 2012).

Cash benefits, rather than leading to an increase in ‘supported familialism’ – that is to a family member providing care in return for financial compensation – more frequently translates into the development of a ‘commodified de-familialisation’ (Saraceno and Keck, 2011: 387), with families outsourcing care services. De-familialisation does not per se cancel the family’s role in mediating between the state, the care-provider and the care-recipient. Even in those households with a live-in care-giver, several tasks remain the family’s responsibility, such as budget management, supervision and transport (NNA, 2010). *Paid care* cannot entirely substitute for *informal care* and remains highly dependent on the establishment of a relationship between the worker, the person cared for and the latter’s kinship network (Da Roit, 2007).

This marketisation of care has combined with the growing entry of migrants into these jobs. In 2011 nearly 900,000 workers were employed in the Italian care sector: 72 per cent of domestic/care workers were migrants, with women making up 88 per cent (Caritas, 2012). The increase in migrant care labour has been both demand-induced and policy-constructed (Andall, 2000; Cangiano et al., 2009; Sciortino, 2004). Otherwise restrictive national immigration policies are positive towards care-givers, relying on cyclical regularisations of undocumented migrants. Migrant care-givers have become an exceptional and positively regarded category (Kilkey et al., 2010). Nevertheless, the legal framework of Italian migration policies and the restrictive labour market lead to care-givers swinging between regularisation and illegality, with their resulting exclusion from permanent legal titles and welfare provisions (Sciortino, 2004). Binding the renewal of residence permits to work contracts, Italian immigration policies also strengthen the positions of care-givers’ private employers (Anderson, 2007).

Given that the demand for elderly care labour is largely met by cheap, flexible migrant workers (Pasquinelli and Rusmini, 2008), even relatively low-income families are able to afford these services (Alemani, 2004; Bettio et al., 2006; Lyon and Glucksmann, 2008; Näre, 2011). However, we should not underestimate persistent social inequalities in terms of differential access to commodified care services (Saraceno, 2010). Cash benefits are granted in Italy independently of the beneficiary’s income and without any use limitations. Thus, while less affluent families can use them to purchase migrant labour, the system privileges richer families over low-income ones. Less affluent families may be able to access only part-time migrant care labour or be compelled to use cash benefits to cover other expenses.

In this context, ‘commodified de-familialisation’ (Saraceno and Keck, 2011) both produces and reflects changes in the gendered division of work within Italian families. While Italian families are reluctant to delegate the care of elderly relatives to care homes, Italian women are increasingly unable or unwilling to take up these responsibilities (Da Roit, 2007). What implications does this have for men and masculinities? Indeed, changing demographic and policy conditions are ‘reconstructing the nature of family relations and roles, and are likely to put increasing pressure on men as care-givers in the future’ (Kramer, 2002: 4).

## Methodology

The article draws from two wider ethnographic studies on migrant domestic/care workers in Italy, which included interviews with their female and male employers. Fieldwork was conducted between 2005 and 2011 in Rome and Milan. The analysis developed here focuses exclusively on men’s experiences, and is based on semi-structured interviews with 17 male employers.

Our informants belonged to an upper class of entrepreneurs, professionals and managers (lawyers, academics, clinic directors and high-ranking government officers); a middle class of skilled non-manual workers (teachers and civil servants); and a working class of manual workers and non-manual routine workers (factory workers, builders and nurses). This distinction corresponds to the categories (bourgeoisie, white-collar middle classes and urban working classes) commonly used to analyse stratification in contemporary Italy, based on a combination of class of origin and educational attainment, and account for the rigidity of the Italian class system relative to other European countries (Barone, 2009; Schizzerotto, 2013). The informants had different family situations: 10 men were single, divorced or widowed, the rest were married. Living arrangements and working conditions were different. Most upper-class employers recruited full-time/live-in care-givers but resided in a separate house. Working-class and some of the middle-class employers recruited part-time care-givers and lived with the elderly relative. Five employers could rely on state cash benefits.

Our sample includes men aged between their late 40s and late 50s, belonging to the so-called ‘sandwich generation’ (Grundy and Henretta, 2006) – which faces the need to combine work with family responsibilities towards *older* generations and with demands emerging from *younger* generations. It also includes retired employers in their 60s who found it necessary to outsource elderly care in order to take up other responsibilities concerning caring for their grandchildren. The burden of intergenerational care varies according to class. The lower the employer’s income, the higher and more differentiated the need to engage with care requests from different subjects (elderly relatives, children and grandchildren). All of this, as we show below, influences men’s experiences.

We met the informants several times over the 2005–2011 timespan. This allowed an understanding of how family relationships and experiences of care changed over time in our informants’ lives, but also of their changing attitudes towards the researchers. As women from the same majority group, we were at once insiders – in terms of our Italian nationality – and outsiders, in so far as we were interviewing men on the subject of a ‘women’s job’. Men’s attitudes during preliminary interviews were sometimes oriented to ‘saving face’ in front of perceived possible criticism for being careless about family responsibilities. Articulating his initial embarrassment, one informant ironically stated that ‘not only his wife and sister were demanding more involvement, but also women’s academicians were checking that he was doing his homework!’ Upper-class men approached us by emphasising their virtue as responsible kin. Middle- and especially working-class men stressed that their involvement was motivated by financial constraints. Yet subsequent interviews offered space for more relaxed conversations. Exchanging with the informants on common problems in arranging elderly care in our respective families made gender difference less salient. Men adopted less defensive stances and more openly reflected on how their attitude towards care was changing with time and experience.

## Men’s Conjugal Status and the Management of Migrant Care Labour

Current literature suggests that the organisation of care labour tends to be structured around a gendered division of work between men and women, with female employers taking most responsibility for hiring, organising and supervising migrant workers in domestic chores and childcare (Anderson, 2007; Hondagneu-Sotelo, 2001; Kilkey et al., 2013). Our data support these findings. However they also show that, for men, fully delegating the management of care-givers to female relatives appears to be more difficult than delegating the supervision of domestic workers and child-carers. The interviewees stressed how the worsening of the elderly relative’s health required them to reconsider their role and routine in the home. Further, our data show how men’s involvement in care is highly influenced by family circumstances such as conjugal status and kin relationship with the elderly person.

Men come to be involved in elderly care in two ways. The first refers to men’s acknowledgement of the need to share organisational tasks with female relatives and enter into a management relationship with the worker. Like the female employers interviewed by Hondagneu-Sotelo (2001), men felt the responsibility of being involved in the selection of a trustworthy care-giver. Alongside the need for ensuring quality care through the establishment of good working relations, employers also actively reflected on normative ideals of masculinity as family members and on the meanings of being a ‘good’ son/son-in-law. The second relates to male employers backing up the worker’s daily routine by personally accomplishing some physical, psychological and emotional care tasks. In both instances male employers are placed within a triadic relationship involving other family members, the migrant worker and the care recipient and assume a mediating role between the expectations of these different subjects. The ways men engage with tasks of both kinds are highly dependent on their family status which, combined with class position, may produce shifts in the gendered division of work.

For married employers – or those in long-term partnerships – care-giver recruitment initially seems to follow the traditional gendered division of work. Male employers often delegate to women the ‘word of mouth’ work and women’s networks become a source of selection and recruitment. Women are often considered more ‘knowledgeable’ about care needs and skilled at this work. Yet male employers’ degree of involvement in management/supervision work increases if the elderly person is a spouse or a blood relative – parent or sibling – rather than an in-law, and/or if the care-giver resides in the same house as the employers and the care beneficiary. Almost all respondents reflected on their unease over fully delegating care management to their wives if the elderly person was their own parent. The case of Alberto, a married factory worker, indicates how men’s initial disposition towards care changed over time. When we first met him in 2006, Alberto was facing the consequences of his mother’s stroke: he said that his job would not have allowed him to be involved in care-related decisions. Over the next two years, the situation changed considerably. Wanda, his wife, found it difficult to combine her own job, the household management and the supervision of her mother-in-law’s migrant care worker. Alberto was progressively forced to reorganise his life. We accompanied him to his mother’s doctor for visits. He regularly checked the stocks of medicines and care materials with the care worker. He was proud of the fact that, aged 51, he had bought a diary for the first time to note ‘what to remember, buy and do’. Later on he also reflected that, while his initial involvement was more self-imposed as a formal weekly programme, over time he came to appreciate making unplanned visits to his mum. His relationship with the care worker also changed, going from clumsy formality to a relaxed and friendly collaboration.

Scholars working on care and masculinity have noted how men tend to assume the role of ‘care commanders’ (Hanlon, 2012: 37) or to stick to a ‘managerial style’ (Calasanti and King, 2007: 520; Russell, 2001) when engaging with relatives’ care needs. In this portrait, upper-class men tend to withdraw from everyday care obligations by delegating the accomplishment of ‘dirty’ tasks, reasserting power relations in the way the family organises/consumes care work. Our data partly confirm this trend. The attitude of upper-class informants differed considerably from that of working-class employers like Alberto. Drawing from norms related to their professional life and adopting more authoritative behaviour, upper-class employers tried to make sense of their role as unpaid care-givers and employers by negotiating gender (Doucet, 2004). This does not necessarily imply the employer’s physical involvement in care. Among the upper classes, daily collaboration with the care-giver is rare (unlike among female employers, working-class and some middle-class male employers). They were more inclined to reproduce class hierarchies, for instance by emphasising their acquired competencies in supervising the employees or by limiting their involvement to more valued and ‘masculine’ care activities related to medical care, such as accompanying a relative to the doctor with the worker’s assistance.

Nevertheless, while upper-class employers preferred in principle to detach themselves from daily ‘dirty work’, during the interviews they shared a similar concern to that of working-class employers like Alberto, wishing to prevent tensions with their wives/partners over the organisation of care. Giorgio, a lawyer, thus expressed his concerns:

I tended to leave all the searching and evaluation to Lidia, my wife … She has many female friends and they know what to ask, how to understand if she is a good worker … This is a women’s business, is about empathy … isn’t it? As a man, I would feel embarrassed also to ask personal things to this person. (Giorgio)

Then, in a subsequent conversation:

I know my mother, she is cranky and demanding: she has a certain life-style and we need to find someone who is good mannered and skilled, Lidia has changed different workers and she is exasperated with my mother’s expectation … so I have to intervene to avoid conflicts … I have to protect my mum … after all, I am her son … but cannot charge Lidia too much … So, the last time I also took part to the interview with the worker, in order to share some responsibilities. (Giorgio)

Giorgio has to mediate between his elderly parent, his wife and the employees, as well as between his filial and conjugal duties. On one hand, he emphasises the appropriateness of women’s networks as a valid way of evaluating a suitable worker, by depicting the tasks associated with recruitment as ‘feminine’. On the other, his filial duties are reasserted through a more active engagement with the organisation of care labour. Kilkey et al. (2013: 97) note how the modernist British model of the ‘active and nurturing father’ is key to the construction of models of upper-class masculinity among professionals employing migrant handymen. This does not necessarily disrupt the gendered division of work within the couple since the management of child-carers remains the mothers’ responsibility. For the upper-class employers we interviewed, engagement with the ‘caring son’ model partly translated into specific demands in terms of the workers’ skilfulness or well-mannered behaviour. These demands were deemed essential to maintain a professional class lifestyle and domestic decorum. In this light we should interpret Giorgio’s concerns about a worker who could meet his mother’s refined standards.

As male employers progressively engage with care labour management, however, this engagement brings about considerable changes in the gendered division of labour. For Giorgio, involvement in care labour management is crucial for avoiding conjugal conflicts. While Giorgio, like other upper-class employers, rarely showed the kind of commitment that characterised working-class employers like Alberto, his attitudes did change. During our first meetings he appeared to focus mainly on his career, while at subsequent meetings he mentioned postponing or cancelling important work trips due to his mother’s worsening condition: ‘For the moment I had to slow down with the work, as I cannot travel very often like before, because I need to be available for emergencies’ (Giorgio, interview in 2010).

Both upper- and middle-class informants recognised that care organisation is ultimately a ‘shared duty’ between wives and husbands, particularly when the care-beneficiary is the man’s parent. Significantly, upper-class employers considered their full withdrawal from care management as demeaning to their wives’ class status – and indirectly, their own.

One important aspect to consider when dealing with employers’ class difference relates to differential access to housing possibilities and to full-time employment of migrant workers. Detachment from the daily care routine is often ensured by the possibility of living separately from the elderly parent and the live-in worker. While among middle-class informants the elderly relative often owned an apartment, generational considerations required some employers to transfer this to their children. Giovanni, a school teacher, brought his mother to live in his flat. He left his mother’s apartment to his daughter. Due to her precarious work situation, she could not afford one herself.

For working-class families, frequent cohabitation with the elderly relative and the fact that care-givers could be hired part-time made the avoidance of care responsibilities more difficult. Dario, a retired factory worker in his mid-60s, decided to invest the cash benefits his paralysed mother receives partly in hiring an Ecuadorian woman to help him. This decision was taken following his own heart attack in 2005:

My wife Mimma has to help our two daughters with their children. After she retired she took up the new job as grandmother! Kindergartens are too expensive and we need to help them. So we needed someone to clean and dress my mother, I cannot do it alone and Mimma comes back very late in the evening and has to cook and clean. Mimma prefers to clean the house herself rather than washing my mother, she gets very depressed with it. So when the woman comes I tell her what to do. I buy the products and medicines for injections and I assist her to feed my mum. She comes here every day for two hours … that’s all I can afford … so I told her that she needs to be on time and to do her job without wasting time on other issues. I try to be always there, to be sure that everything is done properly. (Dario)

This situation was common among our working-class informants. The necessity of covering different care needs across generations using a limited budget intertwined with the reworking of gendered relations between the couple and a resulting shift in the division of work. Dario’s wife preferred to engage with more positively valued care of their grandchildren and with the emotion-free task of cleaning the house: she withdrew from providing physical personal care to her mother-in-law. The daily care was outsourced to a migrant worker, whom Dario was responsible for supervising.

Involvement in care labour was more intense in those instances in which the man had to take care of his wife. David, a retired teacher in his mid-70s, supported his wife Giulia, affected by Alzheimer’s disease:

Philomena comes twice a week. We agreed that the first thing is to bath her and put her in new clothes. Initially I thought I should have hired a man but then I realised that Giulia would not feel comfortable with this … neither I would be. Philomena knows how to take care of a women’s body, she is delicate … if you take a migrant man he might be rude … but of course I have to help her to hold the body while she is washing and dressing Giulia. It was not easy at the beginning, it was painful … is like realising every time the conditions in which she is … but with the time I learned how to cope with it. Now, sometimes I even think I need to do this in order to feel I am still her husband … that I am there. (David)

Russell (2007: 11) highlights the importance of considering those ‘stories of adaptation, transition and commitment’ in men’s care trajectories, in which care-givers’ responsibilities ‘expand beyond instrumental tasks to personal, touching ones’: these stories reveal how care-giving becomes internalised as an ‘integral identity marker’ (2007: 12). While questioning David’s initial desire for detachment, adapting to the daily routine of bodily care eventually became integral to his emotional attachment to his wife. This was also informed by constructions of migrant men as inappropriate for the task. While a male worker is in principle considered more suitable for heavy tasks – potentially discharging David from care duties – the desire of protecting Giulia’s intimacy as well as the association between migrant masculinity and roughness oriented him towards recruiting a female worker and personally undertaking some emotionally charged tasks.

## Single Men and the Management of Migrant Care Labour

Attaining a masculine ‘care commander’ (Hanlon, 2012) role is equally fraught with dilemmas for unmarried informants, who must engage with the practicalities of recruiting/supporting a care-giver. Single status or events like divorce or widowhood may compel men to reconsider their detachment towards active involvement in care work. Compelled to engage with the ‘dirty work’ of care, by engaging with physical and/or bodily hygiene tasks, they deviate from a ‘masculine’ model of care work engagement, focused on ‘instrumental tasks’ (Russell, 2007). Giovanni was trained by the migrant worker on how to perform these ‘dirty work’ tasks during weekends or when she was on leave. This situation was frequent among working-class employers, who, unlike the upper-class informants, could not afford full-time workers or holiday cover. Engagement with care work both resulted from and produced new understandings of gender and relationship to their kinship network for these men.

Unmarried government officer Ruggero, 58, stressed how the impossibility of relying on a female relative transformed his initial refusal to engage with the organisation of care labour:

It is something I had never considered … I was not married but my sister was looking after our father … then she moved to the countryside. So I had to find someone through a recruitment agency … and they arranged three interviews. Then I chose one, a woman from Senegal because she was strong and my father is heavy to carry. I did the entire work for a contract, the residence permit … and now I visit my father twice a week … I mean it is a learning process. (Ruggero)

Long-term single status induced some men to organise care around the support of a female relative, usually a sister. Yet changing family conditions and needs prompt employers to enter into an ‘unexpected’ learning process. Our informants also recognised that unequal distribution of care work between siblings is a potential source of conflict. In subsequent interviews Ruggero told us that his sister’s move was only one reason for his involvement. She had questioned Ruggero’s ‘selfishness’ and disinterest in their father’s condition. Interestingly, Ruggero’s accommodating reaction combined reflections about his filial duties with considerations about his vulnerability as a single man:

I realised that she was right … but also I wanted to avoid conflicts because I am alone … and they are the only relatives I have. So it does not feel good to break up with them, at my age this is even more important. (Ruggero)

Like Ruggero, single, childless men in their late 50s and 60s tend to associate their present role as care worker employers with the prospect of becoming care beneficiaries themselves in future, leading them to question their gendered roles within the wider kinship network. Interestingly, this sense of vulnerability characterised most of our elderly single informants across class difference.

Financial considerations play another important role in the distribution of care work between single male employers and their kin. In middle-class and working-class families struggling to combine different care needs, the full delegation of care to a sister or brother can cause tensions. Pietro, a widowed nurse, increasingly shared care work with his brother and sister after they pointed out that they were meeting most of the expenses (related to the hiring of migrant care worker and to medical treatments), while also having to maintain their own families. Since Pietro had no other family-related expenses, they asked him to care for their mother for at least six months a year.

Ruptures in family life also require men to rethink their position within the family. Before his divorce, Gianfranco, an academic, relied on his wife Sara to supervise care for both their mothers. When we first met him in 2007, he seemed uninterested in sharing tasks with his wife. He told us that Sara was looking after his mother as his job was very demanding. He saw her at the weekends and was always available for emergencies, but refrained from engaging with daily routine tasks. Yet in 2009, after separating painfully from his wife, his position had moved from a ‘selected commitment’ towards a more complex involvement with his mother’s care needs. In the process, both his weekly routine and his understanding of his family role were transformed. Gianfranco admitted that this had exacerbated conjugal tensions, and reflected on how this made him reconsider his personal history:

Now I understand better my wife … on Thursday I have to leave my work earlier and to rush to my mum’s house to replace the care-giver. I have also found someone for Saturday, but it is not easy to manage two different workers … and then Sunday is my turn … It is OK, I mean it is not easy as men of my generations have not been accustomed to all this, there was always someone doing this for you, isn’t it? (Gianfranco)

In 2010 Gianfranco entered into another relationship but was adamant about not involving his new partner in his mother’s care needs. The new relationship should be built around what he defined as ‘new principles and responsibilities’, in order to avoid possible conflicts. Thus changes in family status can question consolidated gendered asymmetries related to care provision and management.

## Conclusion

Care represents one of the most important equality issues in contemporary societies, and a crucial context where family relations are negotiated by different actors – care-providers, care-beneficiaries and family members (Hanlon, 2012; Russell, 2001; Saraceno, 2010). This article has explored men’s involvement in care as employers of migrant care-givers, to challenge an almost exclusive focus on female employer–female employee work relationships in the IDRL.

Our qualitative analysis considers a limited sample of informants but provides novel insights into the interplay of class and masculinity in the IDRL, opening pathways for further research. It highlights the need to analyse how male employers’ strategies within the ‘private’ domain of the home contribute to constructing class and gender hierarchies.

The outsourcing of elderly care labour to migrant workers is central to reproducing hegemonic masculinity in so far as our male informants are able to withdraw from the ‘dirty work’ associated with daily physical care. A traditional gendered division of work applies to the management of migrant care-givers, with men being often involved in ‘ancillary’ roles if compared with women and undertaking ‘instrumental’ ‘masculine’ tasks. Thus gender norms are reproduced not only through assigning and performing care work, but also through managing it. This is particularly true for our upper-class informants, who shape their involvement around a distinction between managerial tasks and direct care services. In this respect, our data partly confirm existing findings that show a ‘managerial’ approach to care predominates among men with higher income and class status (Calasanti and King, 2007; Russell, 2001).

However, we also note that even for our upper-class informants, the employment of a care-giver does not exempt men from some care tasks, such as providing emergency support or assisting the care-giver. The distinction between the care manager role and direct involvement in the ‘dirty work’ is blurred as our informants find it necessary to engage with tasks which were in principle kept at a distance. Among married upper-class informants, avoiding conjugal conflicts and attaining a masculine model of the ‘caring son’ are crucial in moulding men’s care work involvement. For single male employers, vulnerability related to encroaching age combined with the lack of close female support in pushing them towards care responsibilities. Working-class and, to some extent, middle-class interviewees encountered greater difficulties in attaining a ‘care commander’ role. Among married employers, this was often due to the need to combine different care demands across generations and to the necessity of living with the elderly relative. For single informants, the delegation of care to other family members is often a source of conflicts over financial and time-management issues. In both contexts, the impossibility of relying solely on female relatives leads men actively to support the care-giver and to engage with the ‘dirty tasks’ of care work. In this context, while cash benefits allow non-upper-class men to access migrant care labour, they do not erase class differences arising from the need to combine different care needs with limited resources.

This discussion illustrates the need to distinguish between what we can provisionally define as the *ideology* and the *practice* of men’s involvement in care. At the ideological level, male employers straightforwardly dissociated themselves from the ‘women’s business’ of recruitment and supervision. This attitude often characterised our preliminary interviews. However, during subsequent less formal conversations a more nuanced picture emerged. Men were inclined to share how the need to prevent conjugal tensions, comply with models of active filial/kin duty and avoid isolation from the wider circle of kin led them towards a deeper involvement in care management. Among upper-class men this shift was felt necessary in order to assert gendered models of privileged masculinity. Among middle- and working-class men, care engagement reflected the need to cope with different family expectations and prompted a reconsideration of their role within the family.

Based on this analysis, we suggest that the recruitment of a migrant care-giver should be conceived as a critical moment in men’s biographies as well as household organisation. Male employers have to consider family equilibrium, possible conjugal tensions and the need to combine different care responsibilities with professional duties. Our analysis also suggests that class and status are crucial in shaping the terms under which a family can afford to access migrant care labour as well as in moulding men’s role as employers.

## Notes

1. We use Parreñas’ (2001) notion here and understand ‘reproductive labour’ as encompassing moral, emotional and material care addressing the needs of dependent individuals (children and adults). This labour is key to social reproduction, defined as ‘the array of activities and relationships in maintaining people both on a daily basis and inter-generationally’ (Nakano Glenn, 1992: 1).The notion of reproduction has emerged in the 1970s in feminist debates over the relationship between capitalism and patriarchy: it refers to both biological reproduction and the social reproduction sustaining people’s lives that is needed by all production systems (Truong, 1996).
